# Editorial: Slaughterhouses as Sources of Data for Animal Health Intelligence

**DOI:** 10.3389/fvets.2018.00332

**Published:** 2019-01-08

**Authors:** Flavie Vial

**Affiliations:** Epi-Connect, Skogås, Sweden

**Keywords:** slaughterhouses, meat inspection, animal health surveillance, animal welfare, food chain, food safety

The value of meat inspection as an animal health and food safety surveillance tool has been recognized by a number of agencies and organizations, including, the European Food Safety Authority. Because most food animals would eventually be slaughtered, slaughterhouses are considered a key element along the food production chain in which pathological data, information on the presence of pathogens, drug residues, chemical contaminants, and antimicrobial resistance may be measured. Slaughterhouses therefore represent an important control point for the early identification of potential problems that may have an impact on public health as well as on animal health and welfare.

In this research topic, 25 authors contributed 5 original research articles discussing the various animal health intelligence opportunities offered by slaughterhouse surveillance, its limitations, as well as the types of control actions that may be triggered or promoted as a result of it.

When animal carcasses are judged by a trained veterinarian “unfit for human consumption” during the meat inspection process at slaughterhouses, they are condemned and removed from the food production chain. Such condemnations are not just important for food safety; they are also of great interest to the animal production industry and to the veterinary services. The Identification of condemnation risk factors could have the potential to alleviate the substantial economic losses for producers caused by condemnations and increase livestock on-farm welfare. This identification relies on sufficient data, either at the animal-level or at the farm-level, being collected and/or made available through the slaughterhouses' information system software. Decaudin et al. found that studying the relevant risk factors for whole-carcass condemnation, associated with diseases at the farm of origin, of end-cycle sows is made difficult by the fact that traceability at the abattoir in France is done per batch rather than individually for pigs and that limited information is available at the farm level. This observation is context-dependent as the granularity and volume of data will depend on the type of livestock and the country. In Denmark, pig data at the herd-level was retrievable by Fertner et al. who could integrate three Danish databases, holding information on the purchase of antibacterial drugs, herd demographics, and meat inspection data to look for associations between antibacterial treatment and the prevalence of tail-biting-related sequelae in Danish finisher pigs. Data integration, which requires data sharing agreements and data interoperability along the animal production chain, is key to deriving maximum and actionable intelligence. This is demonstrated by Innocent et al. who combined slaughterhouse data with animal movement and climate data to identify environmental risk factors for liver fluke in cattle in Scotland. Their findings have implications for future predictions of liver fluke risk at the regional and national scales while highlighting the importance of environmental factors in on-farm infection risk.

Environmental factors off-farm, for example during the transport of animals to slaughter, also play a role in animal welfare. Peterson et al. used ambient temperature and humidity data from weather stations near United States slaughter plants to predict the incidence and risk of death among swine in-transit and just prior to slaughter. They discuss how current mitigation measures are often not sufficient to prevent swine deaths due to temperature extremes and represent a significant loss of revenue for the swine industry.

Slaughterhouse surveillance often form an integral part of the passive surveillance system for notifiable diseases. For example in cattle herds, meat inspection is an important component of bovine tuberculosis (TB) surveillance, both in countries with and without official tuberculosis freedom (OTF) status, and often complement the live animal skin testing. Changes of current meat inspection procedures to visual-only inspection procedures have the potential to decrease the herd-level detection sensitivity and to substantially impact on bTB control and eradication programmes in non-OTF countries. Contributing to the evidence base needed for the modernization of meat inspection, Willeberg et al. assess the changes in herd-level sensitivity of abattoir surveillance for TB in Ireland (a non-OTF country) should mandatory palpation and incision procedures be omitted, which would greatly decrease the probability of TB-like lesions being observed.

The articles published in this research topic illustrate how the integration of slaughterhouse data with other data collected upstream in the food chain represents a powerful approach to quantifying disease risks in production animals. The results from these studies offer producers actionable (although frequently production system specific) insight into ways meat inspection outcomes could be optimized from the farm. Also highlighted is the part played by other actors in the food production chain, such as transporters, who are less often targeted by interventions aimed at improving animal welfare. Currently being modernized in Europe through a push for less invasive and time-consuming procedures, meat inspection remains nonetheless a central part of most, if not all, European countries' passive surveillance systems. While its primary aim continues to be to protect public health by ensuring that minimal hazardous material enters the food chain, the evidence-base for its utility for animal health surveillance continues to grow with this research topic.

## Author Contributions

FV came up with the idea for this Research Topic and wrote the Editorial.

### Conflict of Interest Statement

The author declares that the research was conducted in the absence of any commercial or financial relationships that could be construed as a potential conflict of interest.

